# Contemporary management of minimal invasive aesthetic treatment of dentition affected by erosion: case report

**DOI:** 10.1186/s12903-019-0807-4

**Published:** 2019-06-21

**Authors:** Philippe Boitelle

**Affiliations:** Department of Prosthodontics, Surgeon Dental Faculty, University Lille – CHU de Lille, Lille, France

**Keywords:** Erosion lesion, Biomimetic, Aesthetic, Prosthetic rehabilitation

## Abstract

**Background:**

The paradigm shift obtained with new dental materials permits minimally invasive dentistry, by following a biomimetic approach. Erosion increasingly affects the adult population through dental substance loss by acid attack. Oral rehabilitation is often extensive and requires careful mouth examination and treatments codified in the literature.

**Case presentation:**

This clinical report proposes a reasoned approach to erosion treatment for a 39-year-old male patient presenting several old fixed prostheses. These old restorations are all of correct quality and are retained. The temporomandibular joint was free from disorder. Only defective reconstructions are remade together with eroded teeth, according to a three-step technical protocol. In the first step, mock-up manufacturing is performed which occlusal vertical dimension increased to 1 mm provoking passive dental overeruption to the second and third molars. In all, one ceramic crown was remade, and two ceramic onlays and a resin composite were integrated on the posterior teeth. The last step consisted of palatal veneers on the maxillary incisor and canine, and an aesthetic resin composite on the incisor edge. After these treatments, regular assessments were carried out at 4 months then at 6 months with visual, photographic and radiographic examinations.

**Conclusion:**

The present dental care philosophy is to preserve dental tissue as much as possible, even in large erosion cases, and to respond to the aesthetic and functional expectations of the patient. This methodology requires a thorough evaluation phase, compliance with the protocol and regular patient follow-up.

## Background

The evolution of the constituent materials of prosthetic restorations and their assembly systems have led to a real paradigm shift in the field of fixed prostheses. Contemporary dentistry has freed itself from the principles of preparation and mechanical retention, which are always unavoidable for conventional fixed prostheses, causing the decay of the dental organ [1, 2]. The challenge, according to Magne et al. [3], is the maximum preservation of dental tissues by implementing this minimally invasive dentistry and replacing, almost exclusively, the lost dental tissues [4–7]. Since, indications relating to partial restorations have been considerably extended to cover even extreme cases of substance loss [2]. The biomimetic approach guides us towards reasoned and carefully thought out dental preparation based on the scrupulous analysis of the location, the architecture and the volume of the lost substance [8].

Dental erosion is now considered a public health problem. The prevalence of tooth structure loss is between 25 and 30% of the adult population [9, 10], and this rate increases with age because the effects of wear accumulate over the course of a lifetime [11]. The excessive intake of soft drinks is one of the etiological factors regularly observed. Clinical diagnoses are based on the compilation of index and use classifications [12]. When tooth brushing is performed well, the risk of caries is limited but teeth are always exposed to acid attacks. Wear can affect many teeth, leading to a decrease in the occlusal vertical dimension (OVD) and the supraeruption of teeth [13–16]. Full-mouth rehabilitation may be required in cases of severe erosion without early care. Clinical activity shows that old reconstructions next to worn teeth in certain patients are in relatively good condition. The “three-step technique” protocol, described by Vailati et al., has demonstrated that additive dentistry that minimizes iatrogenic acts is possible [17–19]. The daily application of all these guidelines must be pragmatic and carefully considered [20]. Indeed, given that these existing excellent restorations simply should not be remade, the treatment therefore consists in restoring them by adding material to the eroded teeth.

This article illustrates a clinical situation in which a case of dental erosion was addressed using the principle of additive dentistry and the three-step technique in patients presenting several old fixed prostheses.

## Case presentation

**Fig. 1:**
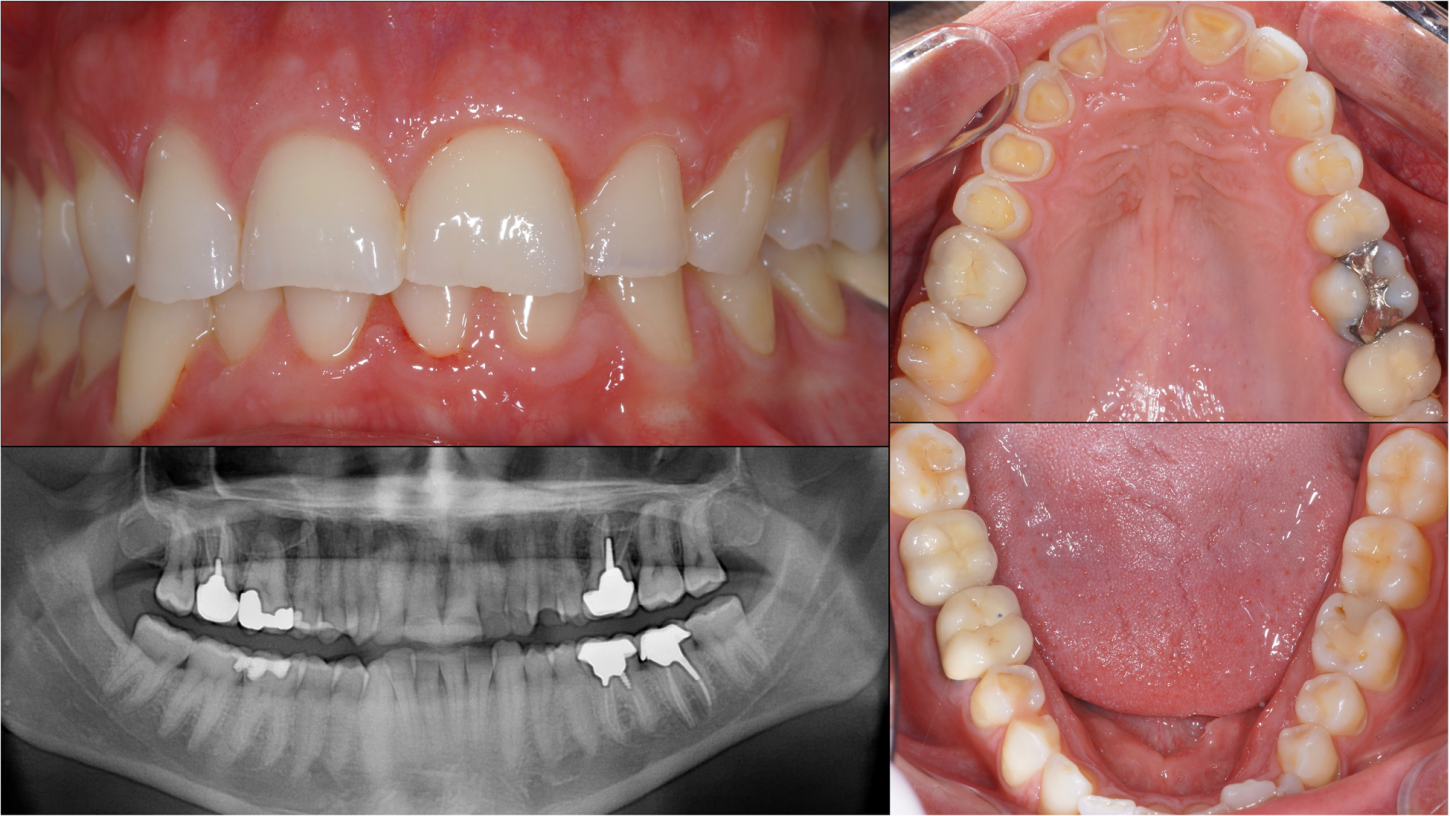
Clinical examination and Panoramic radiographic of the initial dental status

**Fig. 2:**
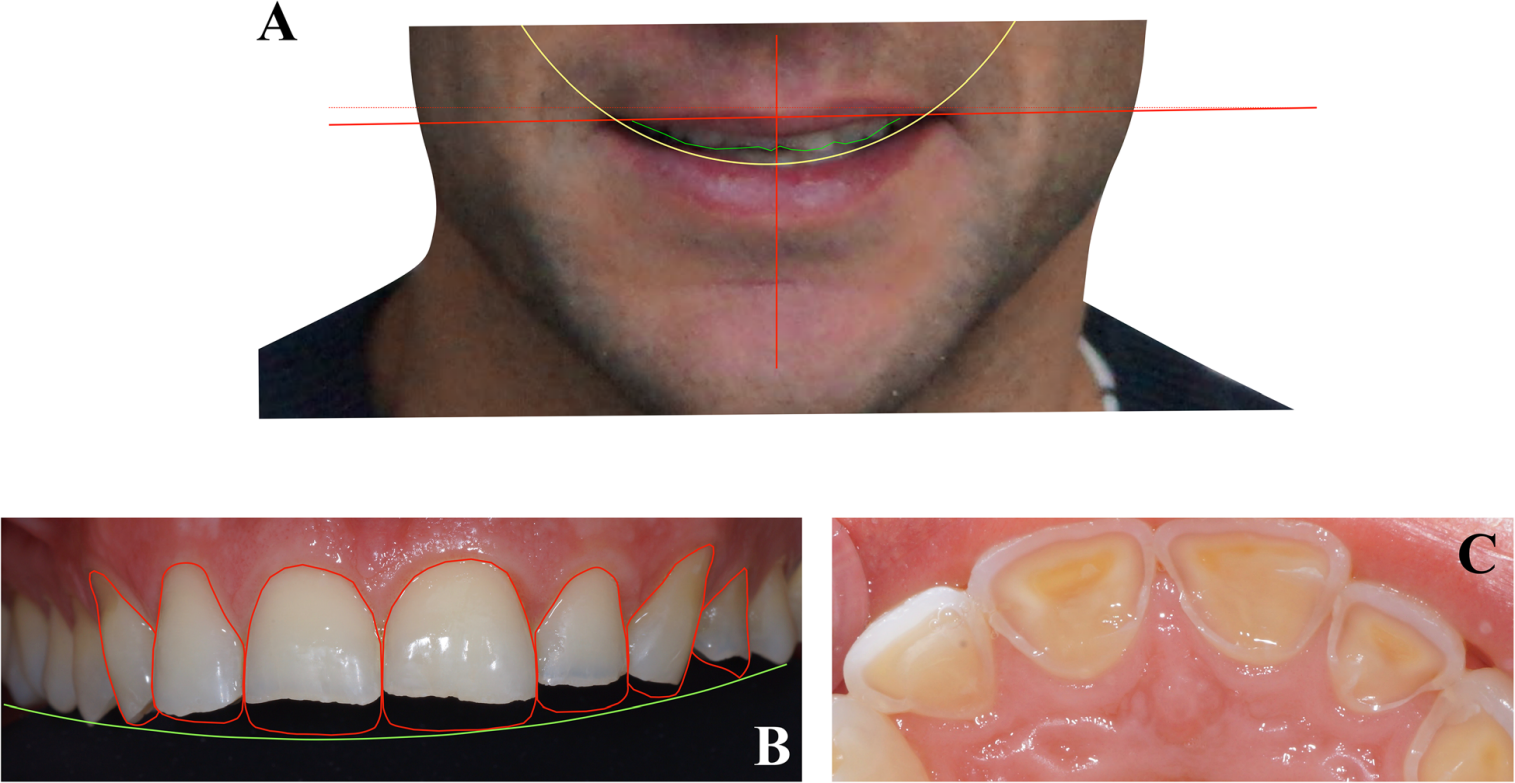
Clinical observations. **a** Facial aesthetic analysis; **b** Analysis of the teeth shape; **c** Erosion level of maxillary incisors

A 39-year-old man visited the dental clinic due to the evolution of his worn teeth, in particular the maxillary incisors. His medical history revealed massive soft drink consumption. Clinical and radiological examinations showed the presence of amalgam at the maxillary right first molar, and resin filling with carious recurrence at the maxillary right first and second molars. Moreover, the mandibular left first molar was subject to carious recurrence below its metal-ceramic crown, requiring its reconstruction (Fig. 1). The loss of non-carious substance affects the maxillary incisors, canines and pre-molars. Severe erosion of type “grade 2” was detectible, with the loss of enamel and dentin surface exposure. The occlusion study underlined that the prosthetic space left by substance losses was not sufficient to obtain the necessary thickness of reconstitution materials. The examination showed no pain during temporomandibular joint and muscular palpation. The patient didn’t report elements suggestive of bruxism. However, the necessary increase in the OVD was estimated at 1 mm inter-incisal. Facial and dental aesthetic analysis revealed no facial asymmetry and no deviation of horizontal facial lines. Analysis of the shape of the maxillary incisors confirmed the loss of substance at the free edge of the incisors and canines, which was the cause of the disturbances of the curvature of the aesthetic frontal curve (Fig. 2).

In this clinical context, the complete rehabilitation of the mouth was proposed to this patient. After discussion with the patient, the decision was made to take a very conservative and reasoned approach to the treatment. The integrity of the residual dental tissues was preserved using adhesive techniques favouring minimalist preparations and restoring the substance losses, by adding the materials composing the restorations [17]. The increase in the OVD to 1 mm was stabilized by the restorations and by passive dental overeruption [13, 14]. All these treatments were to be accompanied by stopping soft drink consumption.

**Fig. 3:**
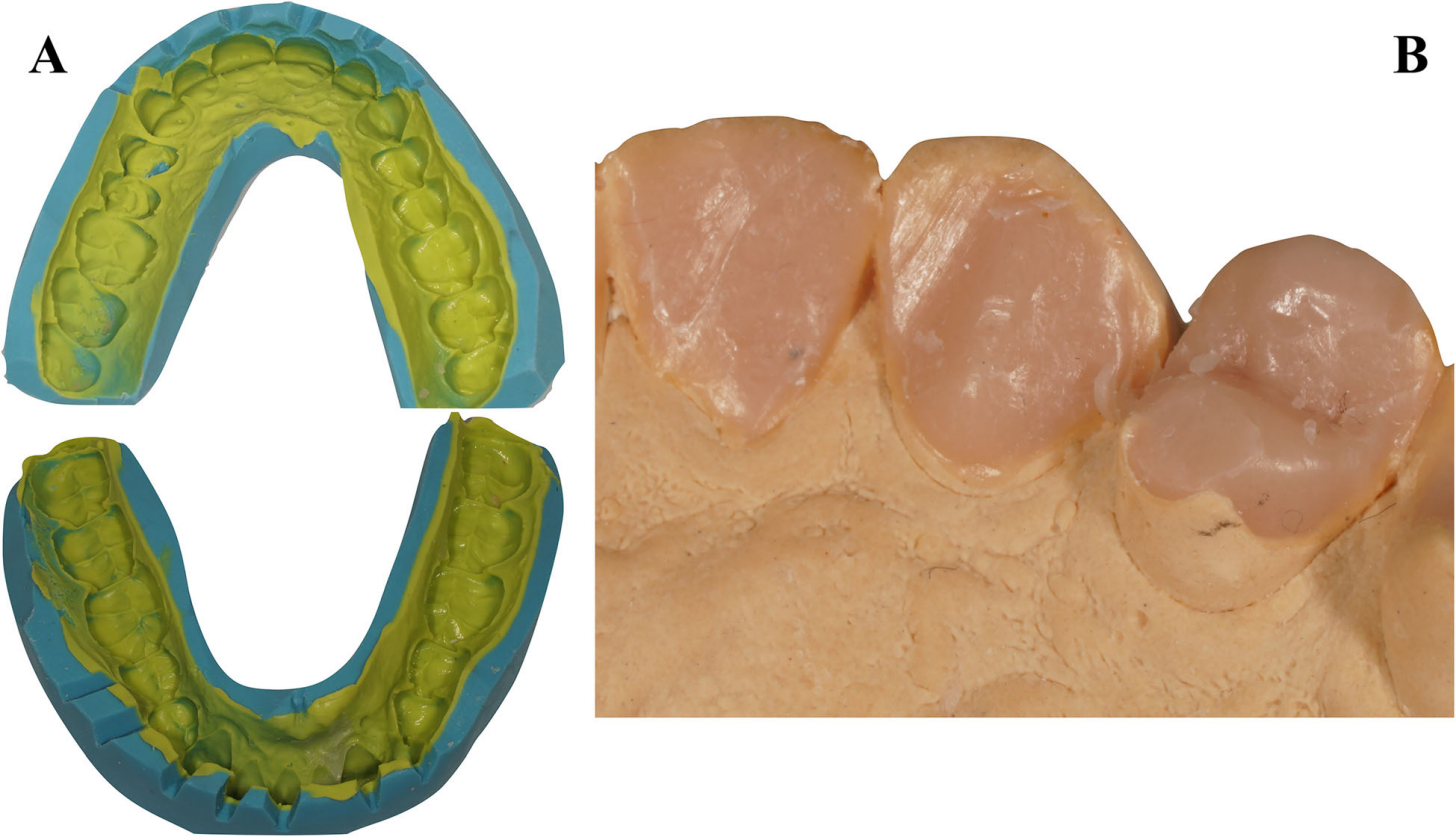
**a** Maxillary and mandibular silicon key; **b** Maxillary wax-up

**Fig. 4:**
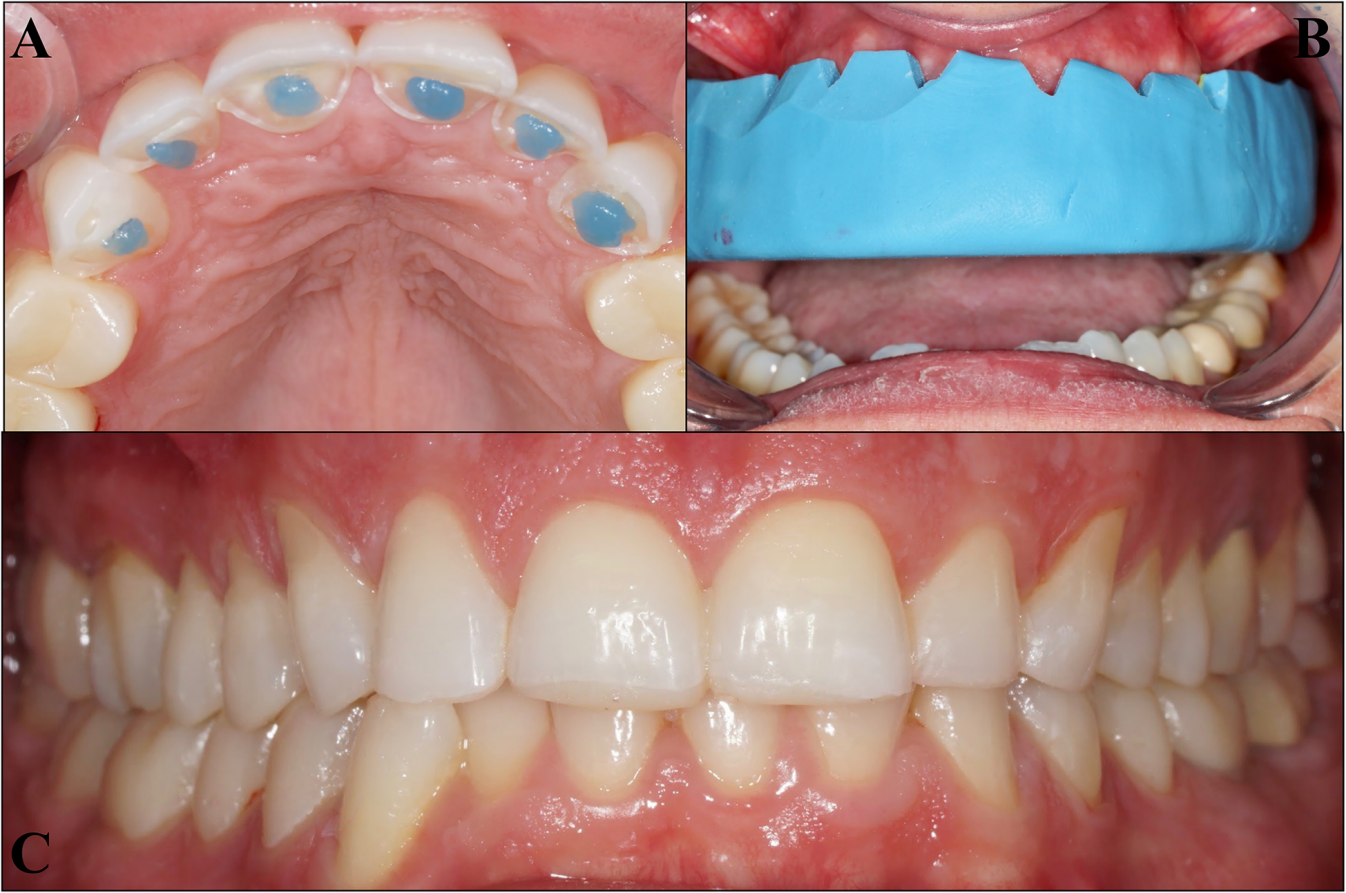
Realization mock-up steps. **a** etching spot; **b** Resin application with silicon key; **c** Full mock-up finish

**Fig. 5:**
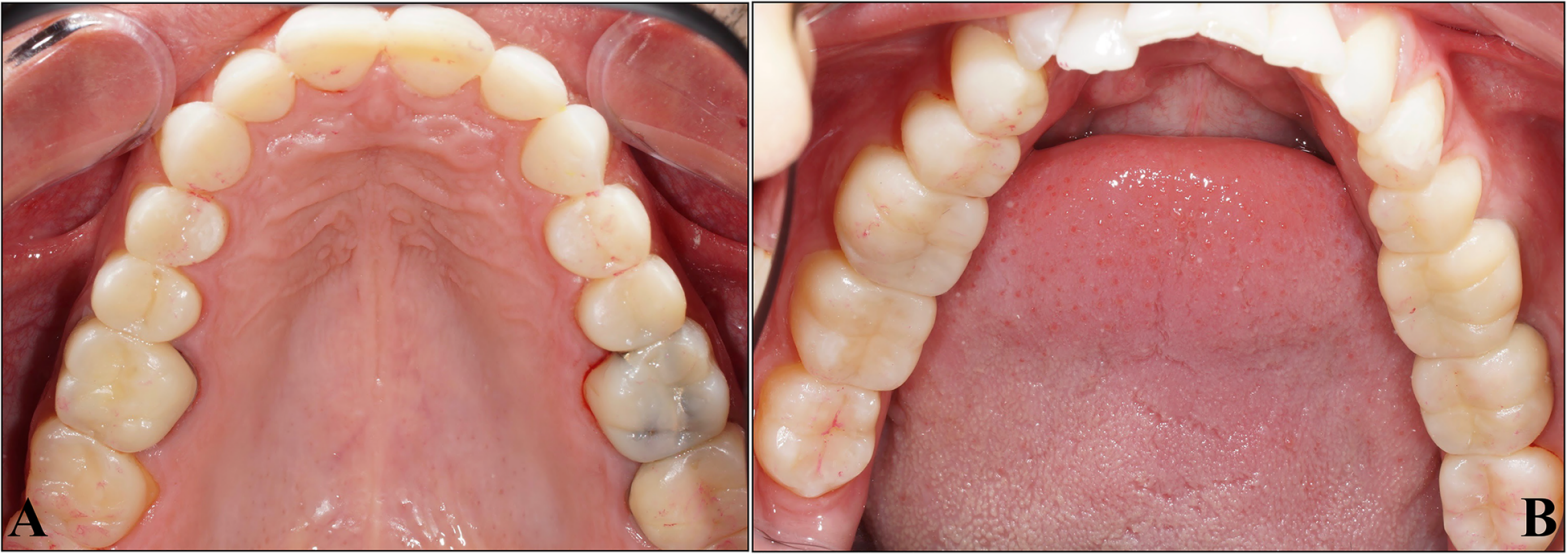
Photography of mock-up: **b**: maxillary arch; **b**: mandibular arch

**Fig. 6:**
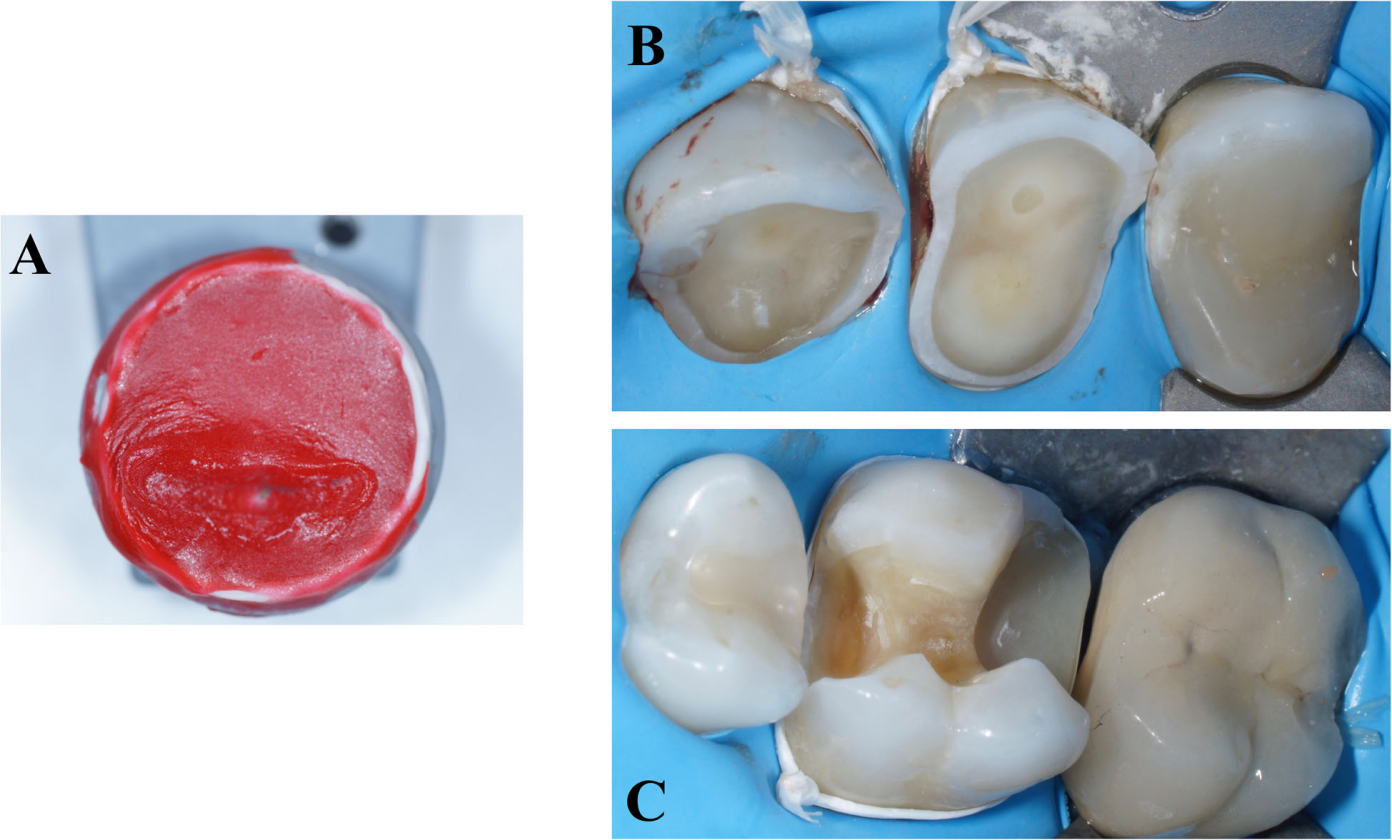
**a** Incisor table personalized; **b** Onlay positioned on the second maxillary left premolar; **c** Onlay preparation on the first maxillary right molar

The treatment plan was performed in three steps. The first clinical appointment was dedicated to maxillary and mandibular impressions (Hydrocolor 5; Zhermack, Badia Polesine, Italy) and the facial arc (Facial arc Quick; FAG Dentaire, France). The increase of the OVD was obtained on the semi-adjustable articulator (Quick Master 1000 B2; FAG Dentaire, France) and a bite wax recorded in centric relation. The first laboratory steps consisted in also making a wax-up of the OVD at + 1 mm on the anterior guidance pin. The technician performed a full-mouth wax-up in three steps (premolar/molar sector right and left; incisor-canine sector), excluding the second and third molars. For each wax-up step, a silicone key (Zetalabor and Zetaflow; Zhermack, Badia Polesine, Italy) was fabricated (Fig. 3). Thus, a total of three-silicone keys per arch were prepared. This technique ensured optimal repositioning and thus high fidelity with the wax-up. On the second clinical appointment, each silicon key loaded with Bis-Acryl-resin (Luxatemp Star, DMG Fabrik GmbH, Germany) was positioned in the patient’s mouth to build the full mock-up. This Bis-Acryl-resin presented a high breaking and bending tolerances increasing mock-up’s clinical survival No teeth were touched during this clinical visit. Only etching points (Gel Etchant; Kerr Dental, California USA) were applied to obtain better adhesion and durability of the mock-up (Fig. 4). A second mock-up visit enabled clinical validation of the aesthetic, phonation and occlusal functions (Fig. 5). The patient and his family validated the new incisor morphology. Anterior guidance, teeth occlusion and the increase of the OVD were confirmed by the integrity of the mock-up and the conservation of the occlusal contact points. The absence of the mock-up at the level of the second and third molars caused an inocclusion that was compensated by teeth overeruption after 3 months of wearing. Once the new occlusion was confirmed, mock-up impressions were made to prepare a personalized acrylic resin incisor table (Pattern Resin LS; GC Corporation, Tokyo Japan) on the articulator (Fig. 6 a). In this temporary time, patient involved a regular oral health check-up to certify the good dental and temporomandibular health.

The second step started by restoring the posterior teeth, notably by the left mandibular quadrant. After endodontic treatment, an inlay-core was cemented on the mandibular left first molar. The mandibular left second premolar was restored with resin composite filling (Tetric EvoCeram; Ivoclar Vivadent AG, Schaan, Liechtenstein). Then, the final preparation of the molar was performed and impressions were made with a two-step putty-wash technique using addition silicone impression material (Express 2 PentaPutti and Express 2 Ultra-light BodyQuick; 3 M Espe™, St Paul, Minnesota USA) and casts were poured using type IV hard stone (Fujirock EP; GC Corporation, Tokyo Japan). The provisional crown was produced using a silicone key made from an intra-oral mock-up impression. The working casts were articulated and definitive restoration was completed on the model from a base metal alloy (Colado CC; Ivoclar Vivadent AG, Schaan, Liechtenstein) and cosmetic porcelain (VITA VM 13; VITA Zahnfabrik, Bad Sackingen, Germany). Then, resin composite restorations were performed on the right mandibular first and second molars. The two maxillary quadrants were produced in the same way, meaning that the onlay preparation impression was made after preparing the cavity and the resin composite on the adjacent teeth. The ceramic onlay (Emax CAD; Ivoclar Vivadent AG, Schaan, Liechtenstein) was bonded on the left maxillary first premolar (Fig. 6 b) and the right maxillary first molar (Fig. 6 c) (Multilink Automix; Ivoclar Vivadent AG, Schaan, Liechtenstein). Previously, immediate dentin sealing had been placed on the prepared dental surfaces, and the resin composite fillings were placed on the right maxillary first and second premolars and the left maxillary second premolar. The set of adhesive restorations was carried out using a dental dam to ensure the best conditions of isolation (Nic Tone rubber dam; SC expert Tech Solutions SRL, Romania).

**Fig. 7:**
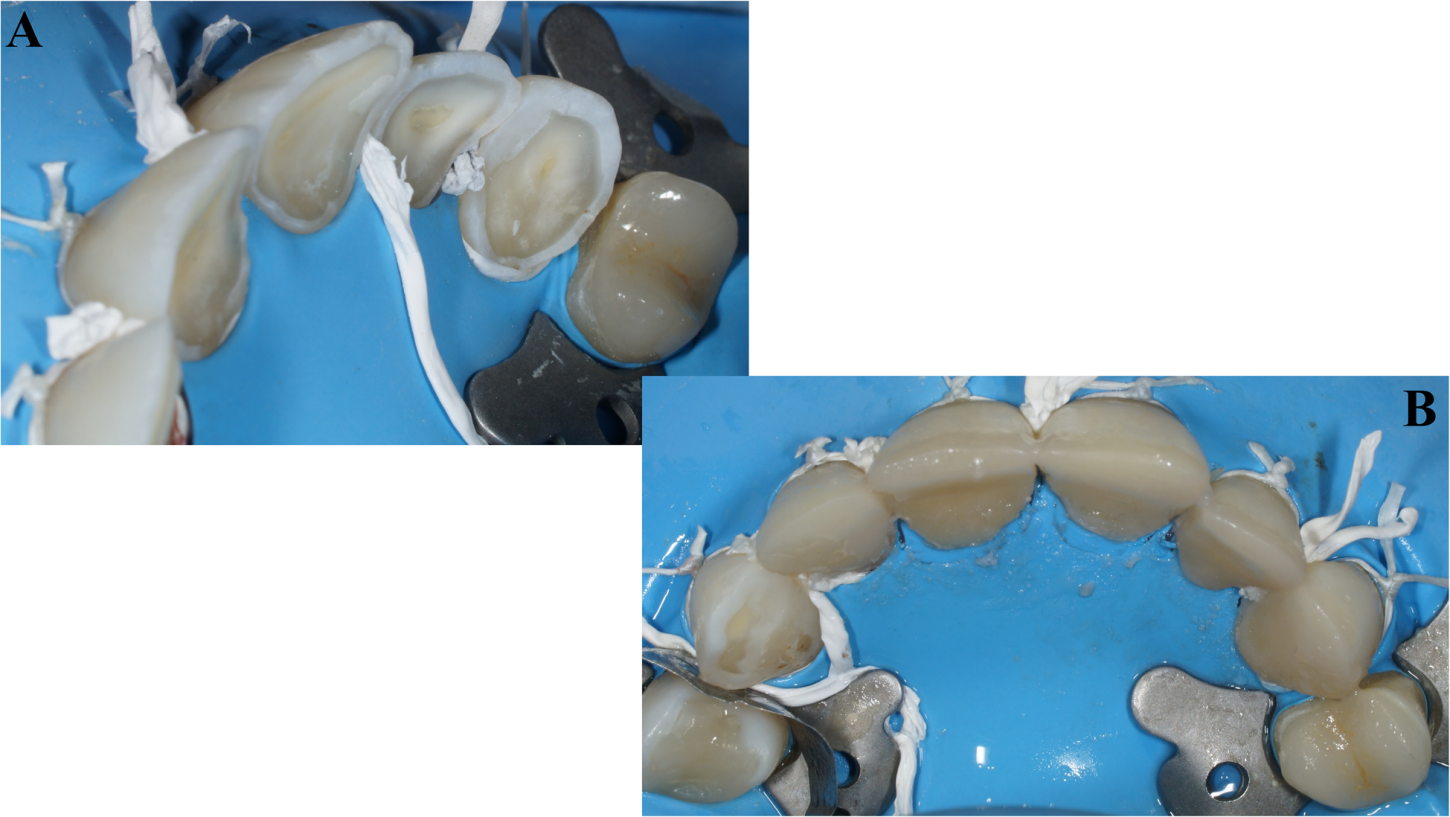
Clinical observations. **a** Facial aesthetic analysis; **b** Analysis of the teeth shape; C: Erosion level of maxillary incisors

**Fig. 8:**
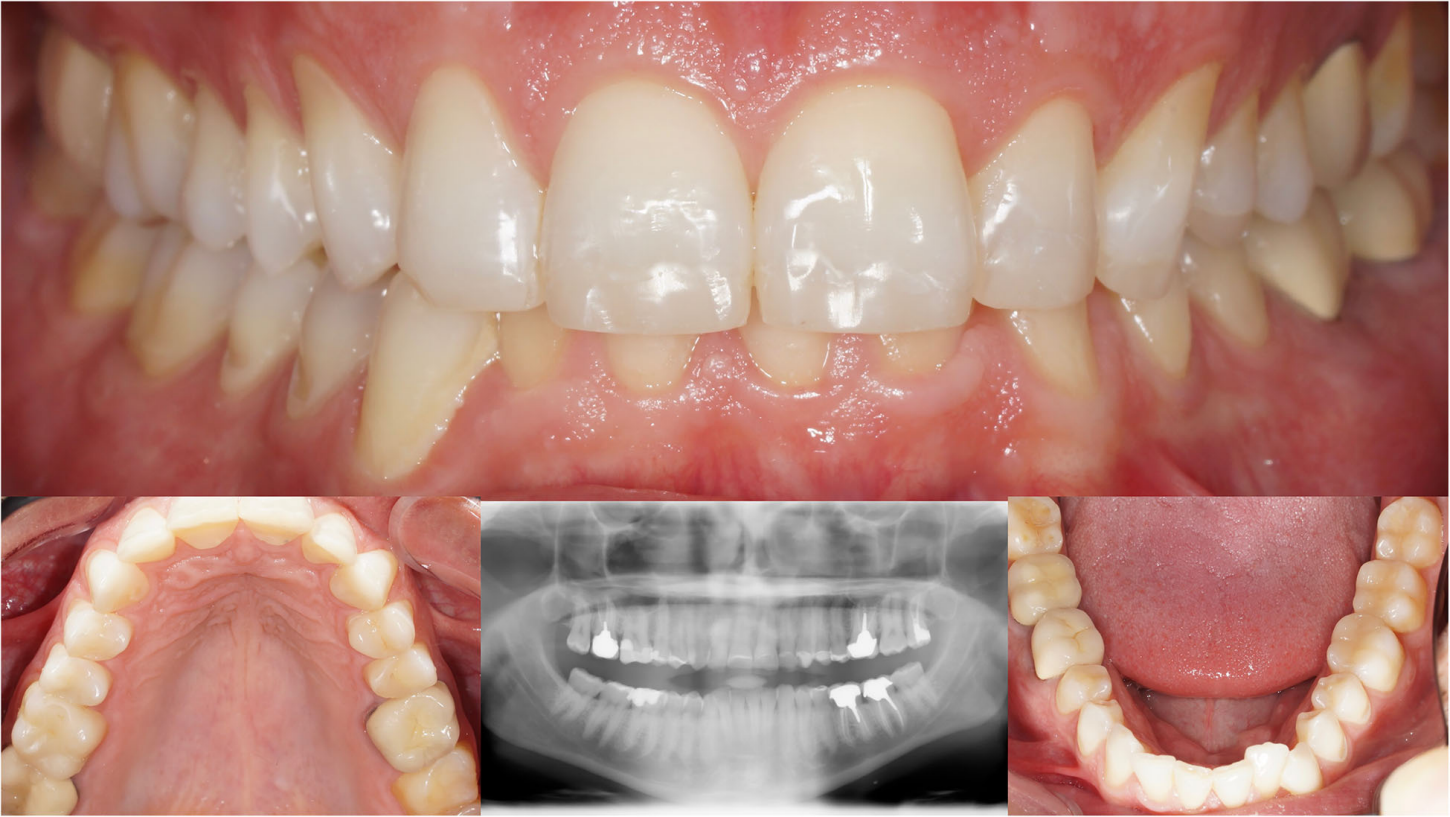
Final situation and Panoramic radiography

The third step consisted of functional prosthetic rehabilitation with palatal veneers and aesthetic restoration of the incisor edge with aesthetic resin composite. The preparations for palatal veneers consisted of simple conditioning of the dental tissue left by erosion. The working casts were articulated, and the incisor table resulted in the manufacture of the palatal restorations with CAD/CAM resin-composite blocks (Lava Ultimate; 3 M Espe™, St Paul, Minnesota USA) and a CAD/CAM system (Fig. 7). The restoration design presented a portion resting on the incisor edge to guide correct positioning during bonding (Multilink Automix; Ivoclar Vivadent AG, Schaan, Liechtenstein). This part was then removed to make room for the aesthetic composite (Estelite Asteria; Tokuyama Dental Corporation, Tokyo, Japan) (Fig. 8). After these treatments, the patient was asked to wear a lower jaw gutter to protect the restorations. The patient acknowledged having improved function and aesthetics and was pleased with the results. Routine clinical assessments were made at 4 months and then at 6 months with visual, photographic and radiographic examinations.

## Discussion and conclusions

The rehabilitation of erosive lesions is one of the most complex treatment procedures described in the recent literature [17–19]. Tooth structure loss frequently affects a large number of teeth and degrades the occlusal and aesthetic functions. These treatments require not only much knowledge of occlusal and aesthetic rehabilitation but also of adhesive dentistry [3]. The aim of the treatments is to replace lost tooth structure with adhesive restorations to prevent excessive loss of sound tissue, in the best occlusal position and by integrating aesthetics [8].

The alternative treatment consisting of rehabilitation without increasing the OVD would have led to protecting the eroded surface with delicate resin composite filling. In this case, the risk of failure of these reconstructions is too great, even when the treatment attempts to serve the patient’s needs and desires.

Previous reports described clinical cases where old reconstructions were remade or no old restorations were present in the mouth [18, 19, 21]. But in daily practice, situations resembling the case reported here are frequent, i.e. the presence of several crowns with the requisite quality of possible adjustment. The limitation of this study was related to the increase of the OVD [16]. In the literature review, Abduo and al. emphasized the safety of increasing OVD. Although discomfort and pain may occur which will disappear after a few days [22]. However, the clinical conditions and a follow-up of the procedures described by the authors should be respected: in the comprehensives prosthodontics rehabilitations, temporomandibular joints in good health, respect of determinants of facial aesthetics (sagittal profile, facial tissues appearance, teeth morphology), and realization a mounting of models for articulator in centric relation. The literature show also that bruxism isn’t a limit to the increase OVD [23]. A thorough evaluation of the vertical dimension is essential. Indeed, occlusion can be validated by the mock-ups [15, 21, 24]. The full mock-up step is a crucial stage of the prosthetic project at both the aesthetic and function levels [8]. Then, using passive dental overeruption can lead to an excessively large increase in the prosthetic marginal fitting and make the cervical part of the tooth root more visible. Occlusal analysis in centric relation relating to the wax-up on the articulator and on the mock-up in the mouth must be carried out properly [20]. Follow-up appointments allow verifying the integrity of the mock-up and the occlusal function. The choice of best mechanic quality bis-acryl-resin is very important to temporary reconstitutions durability. During these sessions, modifications can be made to optimize the functional and aesthetic aspects with intraoral mock-up repairs (i.e., selective grinding, change of dimensions, change of tooth shape with or without a silicone key). If the patient is satisfied and the functional effectiveness is verified, it is then possible to reproduce the anterior guidance obtained with an incisor table. The success of the occlusal adjustments is ensured by this anterior guidance registration.

Dental erosion is a relatively prevalent oral heath disorder in modern society. These enamel and dentin injuries can have severe forms but residual tissues can be used as supports for future adhesive restoration. This paper reviewed a clinical case in which previous dental prostheses were well integrated despite erosive injuries. The dental care guidelines were to allow the maximum preservation of dental tissues and to respond to the patient’s aesthetic and functional expectations. The procurement of prosthetic space allowed selective passive dental overeruption and increasing the OVD to 1 mm. This method requires an evaluation phase, compliance with the protocol and regular follow-ups, all performed efficiently.

## Data Availability

All data generated or analyzed during this study are included in this published article.

## Abbreviations

CAD/CAM: Computer Assisted Design/Computer Assisted Manufacture
OVD: Occlusal vertical dimension
